# Remodelling of a polypyrimidine tract-binding protein complex during apoptosis activates cellular IRESs

**DOI:** 10.1038/cdd.2013.135

**Published:** 2013-10-18

**Authors:** H A King, L C Cobbold, X Pichon, T Pöyry, L A Wilson, H Booden, R Jukes-Jones, K Cain, K S Lilley, M Bushell, A E Willis

**Affiliations:** 1 Medical Research Council Toxicology Unit, Lancaster Road, Leicester LE1 9HN, UK; 2 Department of Biochemistry, University of Cambridge, Tennis Court Road, Cambridge CB2 1GA, UK

**Keywords:** internal ribosome entry, protein synthesis, translation, apoptosis

## Abstract

Post-transcriptional control of gene expression is mediated by the interaction of RNA-binding proteins with their cognate mRNAs that specifically regulate their stability, localization and translation. mRNA-binding proteins are multifunctional and it has been proposed therefore that a combinatorial RNA-binding protein code exists that allows specific protein sub-complexes to control cytoplasmic gene expression under a range of pathophysiological conditions. We show that polypyrimidine tract-binding protein (PTB) is central to one such complex that forms in apoptotic cells. Thus, during apoptosis initiated by TNF-related apoptosis inducing ligand there is a change in the repertoire of RNA-binding proteins with which PTB interacts. We show that altering the cellular levels of PTB and its binding partners, either singly or in combination, is sufficient to directly change the rates of apoptosis with increased expression of PTB, YBX1, PSF and NONO/p54^nrb^ accelerating this process. Mechanistically, we show that these proteins post-transcriptionally regulate gene expression, and therefore apoptotic rates, by interacting with and stimulating the activity of RNA elements (internal ribosome entry segments) found in mRNAs that are translated during apoptosis. Taken together, our data show that PTB function is controlled by a set of co-recruited proteins and importantly provide further evidence that it is possible to dictate cell fate by modulating cytoplasmic gene expression pathways alone.

## Introduction

During pathophysiological conditions of stress including viral infection, amino-acid starvation and apoptosis cells respond by reprogramming protein synthesis.^1^ This is brought about by a reduction in global protein synthesis and, in parallel, the recruitment of specific mRNAs whose protein products are required to respond to the stress or execute a particular cellular process; for example, during apoptosis there is an increase in the synthesis of proteins that are required for chromatin remodelling.^2^

The inhibition of protein synthesis that occurs during cell stress is achieved by modification of the canonical protein synthesis machinery (eukaryotic initiation factors) and cells have therefore developed specialized mechanisms to bring about the recruitment of selective subsets of mRNAs to actively translate ribosomes under these conditions. In general, this is mediated by the interactions of RNA elements in the 5′ and 3′ UTRs of mRNAs with their cognate mRNA-binding proteins/regulatory RNAs.^3^ Many *cis*-acting mRNA regulatory elements that permit selective translation under different conditions of cell stress have been identified including internal ribosome entry segments (IRESs)^2^ and upstream open reading frames^4^ in the 5′ UTR and miRNA-binding sites and stability determinants^3^ in the 3′ UTR. However, relatively few of the *trans*-acting RNA-binding protein complexes that are required to recruit mRNAs to the polysomes have been identified. Interestingly, it has been suggested that a combinatorial code exists where complexes comprising of different subsets of RNA-binding proteins are formed under distinct cellular conditions. It has been hypothesized therefore that such complexes, by controlling gene expression post-transcriptionally would be essential for the full execution of a cellular process.^5, 6^ To address this question under a defined cell stress condition, we studied the role of polypyrimidine tract-binding protein (PTB)-dependent complexes during apoptosis initiated by TNF-related apoptosis inducing ligand (TRAIL). There were a number of pertinent reasons for the choice of PTB and its role in apoptotic cells. First, the known characteristics of PTB would suggest that it could be a central node in a combinatorial RNA-binding protein code as it is a multifunctional mRNA-binding protein with roles in many aspects of post-transcriptional control of gene expression including alternative splicing,^7, 8^ RNA stability, localization^9^ and translation.^10^ Second, PTB is found in large ribonuclear complexes and several known binding partners have been identified, for example, PSF and hnRNPL.^11, 12, 13^ Finally, the IRESs that function during TRAIL-mediated apoptosis are PTB dependent and we hypothesized that such a PTB-containing mRNA-binding complex would interact with these elements.

Our data suggest that there was substantial remodelling of PTB-containing complexes in apoptotic cells concomitant with changes in protein localization. We show that PTB and its interacting partners influence apoptotic rates suggesting that cell fate is dictated by these protein complexes. RNA affinity purification and proteomic analysis identified the proteins that interacted directly with the IRES elements found in the 5′ UTRs of mRNAs that are known to remain polysomally associated during apoptosis. Importantly, there was a large degree of overlap between the proteins that interacted with the IRES elements and those present within the PTB-containing complexes. Taken together, the data obtained from these large-scale screens suggest that during TRAIL-mediated apoptosis there is remodelling of PTB-containing complexes allowing translation of a subset of PTB-dependent mRNAs whose protein products are required to execute this process.

## Results

### PTB-containing complexes are remodelled during apoptosis

To identify the proteins that interact with PTB and whether these changes during apoptosis proteomics-based approaches and western analysis were used.

**Figure 1:**
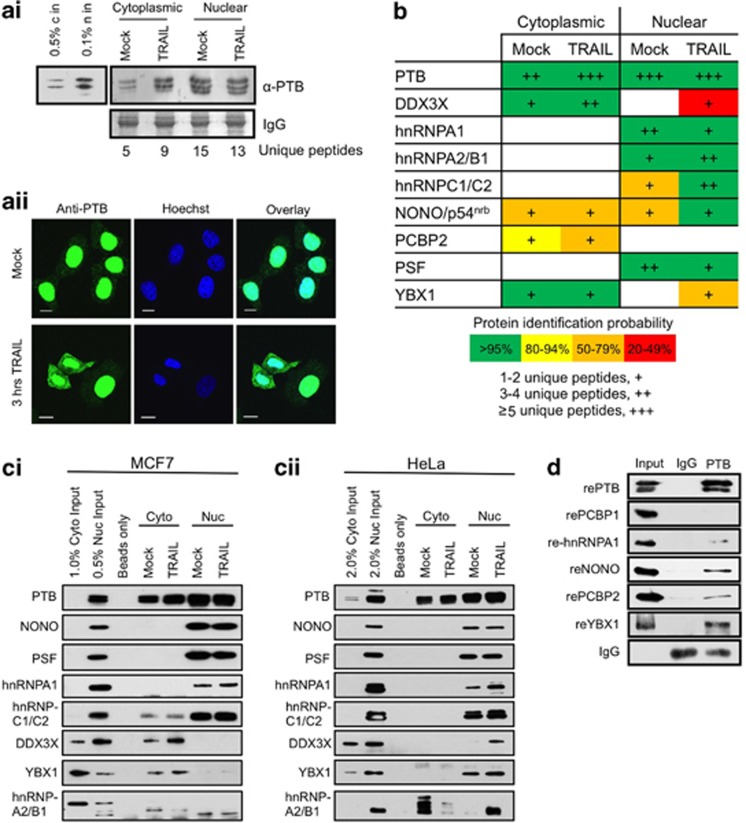
PTB-containing complex that forms during TRAIL-mediated apoptosis. MCF7 cell were induced to undergo apoptosis via treatment with 0.5 *μ*g/*μ*l TRAIL for 3 h, then harvested and fractionated to produce nuclear and cytoplasmic lysates. Mock and TRAIL-treated cytoplasmic/nuclear lysates were used in an anti-PTB immunoprecipitation, which were then analyzed via immunoblotting and LC-MS/MS mass spectrometry. (**a**i) Western blot with *α*-PTB antibody. IgG was used as a loading control from the IP reaction. Number of unique peptides identified for PTB is shown. (**a**ii) Immunofluorescence was used to confirm the relocalization of PTB following TRAIL-treatment. MCF cells±TRAIL for 3 h were stained with *α*-PTB antibody and observed by confocal microscopy. White bar represents 10 *μ*m. (**b**) Table showing PTB-binding partners identified by LC-MS/MS mass spectrometry. A color scale is used to illustrate the number of unique peptides. Number of plus (e.g., +/++/+++) are used to represent the relative abundance of each species detected. (**c**i) Western blotting of the control and TRAIL treated MCF7 cell lysates was performed using antibodies to the indicated proteins to investigate PTB-interacting partners. (**c**ii) The immunoprecipitations of PTB and its interacting partners were then repeated in HeLa cells. Western blotting of control and TRAIL treated HeLa cell lysates was performed using antibodies to the indicated proteins to investigate PTB-interacting partners in this cell type. (**d**) RNA-independent association of the different PTB partners were confirmed by carrying out anti-PTB immunoprecipitations *in vitro* following incubation of recombinant-PTB (rePTB) with rePCBP1, re-hnRNPA1, reNONO, rePCBP2 and reYBX1. Western blot using antibodies to the indicated proteins is shown; IgG was used as a loading control

For proteomic analysis, MCF7 cells were exposed to TRAIL to initiate apoptosis, and nuclear and cytoplasmic extracts were generated from either apoptotic or control cells. PTB and its interacting partners were immunoprecipitated from these extracts and applied to SDS-PAGE. After separation, samples were subjected to tandem LC-MS/MS mass spectrometric analysis; this analysis was repeated on a further two independent occasions. Both the number of unique peptides identified for PTB and the immunoblot analysis show that there was an increase in the amount of PTB in the cytoplasm during TRAIL treatment, and a corresponding decrease in the amount of PTB in the nucleus (Figure 1ai), in agreement with previous data.^2, 14^ These data were confirmed using immunofluorescence and confocal microscopy, and after 3-h TRAIL treatment cells show a marked increase in the cytoplasmic concentration of PTB (Figure 1ai).

Tandem LC-MS/MS was then used to identify putative interacting proteins (Figure 1b and Supplementary Figure S1A). Complexes formed both in the nucleus and the cytoplasm were examined because for some RNA elements it has been suggested that the RNA-binding proteins that control their cytoplasmic activity are pre-loaded in the nucleus.^15^ Immunoprecipitation reactions using antibodies directed against PTB were then carried out on cell extracts obtained from MCF7 and HeLa cells treated with TRAIL to confirm some of these interactions, and moreover to examine whether the complexes formed were present in cells of different origin (Figures 1b and c). In the majority of cases, the interactions identified using tandem LC-MS/MS were confirmed by western analysis (Figure 1ci). Taken together, these data suggest that in the nucleus of control MCF7 cells PTB is present in a complex that includes the RNA-binding proteins NONO/p54^nrb^, PSF, hnRNPA1, hnRNPC1/C2 and hnRNPA2/B1. Changes in the composition of this complex during TRAIL-mediated apoptosis were detected by tandem LC-MS/MS showing there is an increase in the levels of hnRNPC1/C2 and hnRNPA2/B1 (Figure 1b) although no significant differences in hnRNPA2/B1 were detected by western blotting (Figure 1ci). However, both hnRNPC1/C2 and hnRNPA2/B1 were shown to increase with TRAIL treatment in HeLa cell nuclear fraction (Figure 1cii). DDX3X and YBX1 were detected in the PTB complex in the nuclear fraction of the apoptotic MCF7 cells by LC-MS/MS analysis, but this could not be confirmed by western blot, yet these complexes were detected in HeLa cells (Figure 1cii).

In the cytoplasm of the control MCF7 cells, PTB was present in a complex that contains DDX3X and YBX1 and the amount of both of these proteins were increased with the TRAIL treatment (Figures 1b and ci). Neither of these proteins was observed in the PTB complex in the HeLa cell cytoplasmic fraction (Figure 1cii).

RNAse A was included in immunoprecipitation reactions to determine whether the association of these proteins with PTB occurred via direct protein–protein interactions, or indirectly via an RNA intermediate. However, with exception of NONO/p54^nrb^ the interaction of all other proteins with PTB was RNA independent (data not shown).

To confirm that these proteins were able to interact directly *in vitro*, recombinant proteins were generated and incubated at equal molar ratios with rePTB (Figure 1d). The protein complexes were isolated using an anti-PTB antibody, separated by SDS-PAGE and immunoblotted with the antibodies shown. The data showed that recombinant YBX1, NONO/p54^nrb^ and hnRNPA1 interacted with PTB *in vitro* (Figure 1d), confirming the *in vivo* data, whereas rePCBP1, which was not identified as part of the PTB complex, did not interact with rePTB (Figure 1d).

**Figure 2:**
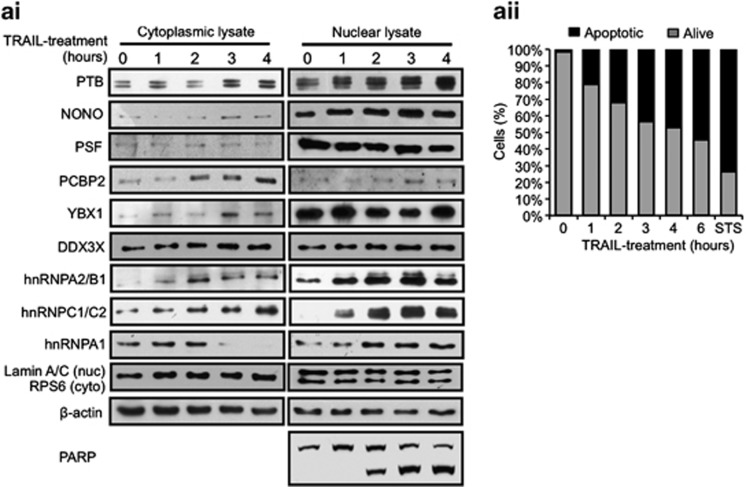
Relocalization of members of the PTB complex during apoptosis. (**a**i) Western blotting of nuclear and cytoplasmic fractionated lysates of MCF7 cells treated with TRAIL over a 4 h time course using antibodies against indicated proteins. RPS6 and Lamin A/C antibodies were used as cytoplasmic and nuclear markers, respectively. PARP cleavage was used to indicate apoptosis and *β*-actin was used as a loading control. (**a**ii) Control and TRAIL-treated MCF7 cells were stained with Annexin V-FITC and propidium iodide at the indicated time points and subjected to FACS analysis. Staurosporin-treated MCF7 cells (STS) served as a positive control at 6 h

The immunoprecipitation data suggest that during apoptosis there was a cellular relocalization of PTB-associated proteins. Therefore, MCF7 cells were incubated with TRAIL for 1, 2, 3 or 4 h, lysed and separated into nuclear and cytoplasmic fractions, applied to SDS-PAGE and immunoblotted with the antibodies shown (Figure 2ai). The data confirmed an increase in the PTB-associated mRNA-binding-proteins YBX1 and DDX3X during apoptosis. There was also increase in the cytoplasmic levels of PCBP2, hnRNPA2/B1 and hnRNPC1/C2 at 4-h incubation with TRAIL (Figure 2ai). HnRNPA1 was shown to relocate from the cytoplasm to the nucleus at 3 h, notably at the same time point where the cytoplasmic concentration of PTB increases, this is of interest because it has been shown previously that hnRNPA1 is a negative regulator of the Apaf-1 IRES.^28^ Many of the proteins identified as part of the PTB-containing complex are known to have other cellular functions, and therefore not all of the changes in location/increases in expression will correlate directly with enhanced association with PTB. To ensure that apoptosis was occurring, cells were analyzed by Annexin V labelling and propidium iodide (Figure 2aii)

### The PTB-containing complex interacts with a subset of cellular mRNAs

**Figure 3:**
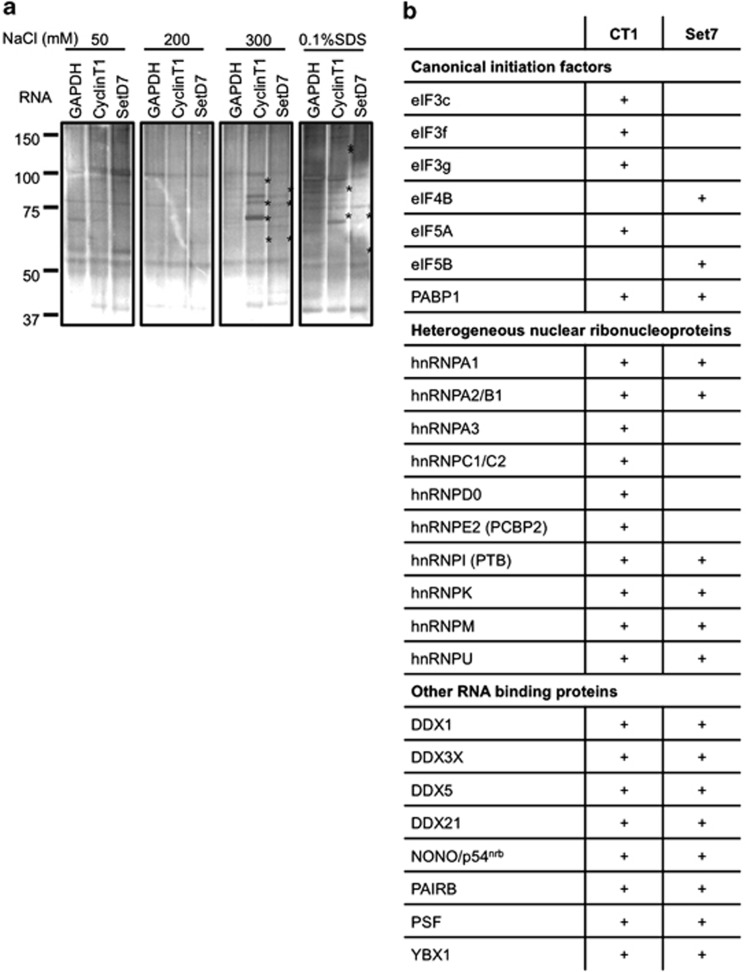
IRES RNA affinity analysis. (**a**) In all, 50 *μ*g of biotin-tagged cyclin T1 and SETD7 5' UTRs or 300 nt RNA of GAPDH coding region was used as bait to purify IRES-binding proteins from HeLa cell lysate in a binding buffer (10 mM Tris pH 7.5, 25 mM KCl, 2 mM MgCl_2_, 0.02% Tween-20, 1 mM ATP, 1 *μ*g/ml yeast tRNA, 1 *μ*g/ml heparin, RNase inhibitor, 1 × protease inhibitor) for 60 min at 4 °C. Streptavidin-conjugated magnetic beads (Invitrogen) were added and incubated for a further 30 min. The bound complexes were washed with buffers that contained increasing salt concentration and eluted samples were applied to SDS-PAGE and stained with colloidal Coomassie blue and silver. Proteins that were specifically eluted from the cyclin T1 or SETD7IRESs, but not the GAPDH are marked (*). (**b**) Proteins bound to each RNA were identified firstly via LC-MS/MS mass spectrometry (Supplementary Figures S2A and B) and then via immunoblotting analysis (Supplementary Figure S2C). The table shows proteins, which were identified as binding to either or both of the cyclin T1 and SETD7 IRESs, whereby a plus indicates binding, in either the mass spectrometry data, the immunoblotting data or both. The proteins included in the table are those found to be both IRES binding and PTB associated

During apoptosis, reprogramming of the translational machinery permits a specific subset of PTB-dependent IRES-containing mRNAs to remain associated with the polysomes including mRNAs encoding SETD7 (SET7/9), cyclin T1, MTG8A, Apaf-1 and cyp1b1.^2^ These mRNAs encode proteins that have a role in the apoptotic process that include proteins that are central to the formation of the apoptosome, for example, Apaf-1,^16^ proteins that modify transcription factor activity leading to upregulation of proapoptotic target gene transcription, for example, SETD7,^17^ and cell cycle regulators, for example, cyclin T1.^18, 19^ Therefore, to determine whether proteins that formed part of the PTB-containing complex also interacted with these IRES elements, RNA affinity purification was used. Biotinylated RNAs were incubated with HeLa cell extracts (both nuclear and cytoplasmic) and the protein complexes isolated (see Materials and methods section). Proteins were eluted, separated by SDS-PAGE and identified by LC-MS/MS (Figure 3a, and Supplementary Figures S2A–C); these experiments were performed on three independent occasions. The data show that a large number of the RNA-binding proteins that were part of the PTB-containing complex also interacted with both cyclin T1 and SETD7 5′ UTRs (Figure 3b) and importantly, with other cellular IRESs tested (Supplementary Figure S2). Thus, the proteomic data show that cyclin T1 and SETD7 interacted, either directly or indirectly, with 14 members of the PTB complex including PTB, hnRNPA1, hnRNPA2/B1, hnRNPK, DDX1, 3X, 5 and 21 and NONO/p54^nrb^, PSF and YBX1 (Figure 3b). Some proteins identified are known to stimulate the activity of other cellular IRESs. For example, hnRNPC1/C2, hnRNPK, NONO/p54^nrb^ and PSF are required for c-*myc* IRES-mediated translation (for reviews, see Komar and Hatzoglou^20^ and Spriggs *et al.*^21^).

### The proteins identified interact with cyclin T1 and SETD7 mRNAs *in vitro* and *in vivo*

**Figure 4:**
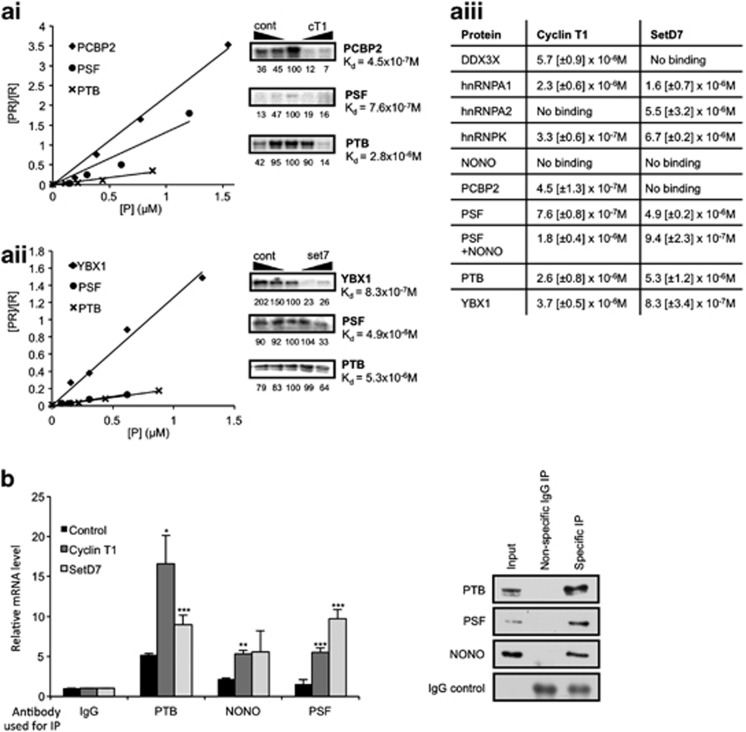
Members of the PTB-containing complex bind apoptotic IRES RNA *in vitro* and *in vivo*. Binding of the identified complex members to the apoptotic IRES RNA was validated by a number of methods. (**a**) UV-crosslinking (right panels) and filter-binding assays (left panels) using ^32^P-labelled cyclin T1 or SETD7 5' UTR. (**a**i) ^32^P-CTP-labelled cyclin T1 and rePTB, rePSF and rePCBP2 or (**a**ii) ^32^P-CTP-labelled SETD7 RNA with rePTB, rePSF and reYBX1 were used in UV-crosslinking and filter-binding assays. Specificity of the binding was demonstrated by increasing the amount of cold competitor IRES RNA (right panel), or nonspecific control RNA (left panel). The UV-crosslinked signal was quantified relative to the central no competitor RNA lane, which was set to a 100. (**a**iii) Dissociation constants for each protein/RNA combination were calculated in the filter-binding assay. A constant level of the ^32^P-labelled probe was used over a range of molarity of each recombinant protein. S.D. is shown in square brackets, and the data are the average of three repeats. (**b**) Binding of endogenous PTB, PSF or NONO/p54^nrb^ to the IRES RNA was shown using RNA-IP with antibodies against the proteins indicated, followed by RT-qPCR using primers specific for cyclin T1, SETD7 or control RNA. 15 cm confluent plates of HeLa cells were lysed in lysis buffer (20 mM HEPES pH7.2, 100 mM KCl, 5 mM MgCl_2_, 1 mM DTT, RNAsin 400 U/ml, 0.5% Triton X-100 and protease inhibitors), incubated for 5 min at 4 °C and centrifuged to pellet the nuclei. The post-nuclear extract was incubated with PTB, PSF or NONO/p54^nrb^ antibodies or IgG (control) coated protein A beads for 1 h at 4 °C. Beads were washed 4 × 30 min with buffer A (20 mM Hepes pH 7.2, 200 mM NaCl, 5 mM MgCl_2_, 0.5% Triton X-100, 1 mM DTT) and 2 × with buffer B (50 mM Tris pH 7.4, 150 mM NaCl, 1 mM MgCl_2_ and 0.05% NP40). Beads containing protein-RNA complexes were isolated and re-suspended in 200 *μ*l buffer B and proteinase K treated for 45 min at 55 °C. The RNA was isolated by phenol/chloroform extraction and quantified by RT-qPCR using primers specific for cyclin T1, SETD7 or control RNA. Results are shown relative to a nonspecific IgG immunoprecipitation. Western blot of the IPs using antibodies against proteins indicated is shown on the right. Significance (*P<0.05, **P<0.01 or ***P<0.005) was calculated using an unpaired two-tailed Student's *t*-test (n=3), error bars represent S.D.

It was then important to determine whether these proteins interacted directly with cellular IRESs (Figure 4 and Supplementary Figure S3) and, where appropriate, the strength of these interactions (Figure 4). Thus, radiolabelled IRES RNA was generated and filter-binding assays were carried out with PTB, PSF, NONO/p54^nrb^, hnRNPA1, hnRNPA2, hnRNPK, PCBP2, YBX1 and DDX3X singly and in some cases in combination (Figure 4a and Supplementary Figure S3A). With the exception of NONO/p54^nrb^ and hnRNPA2 all proteins interacted directly with the cyclin T1 IRES, albeit to different extents. SETD7 IRES interacted with all proteins with the exception of NONO/p54^nrb^, DDX3X or PCBP2, suggesting that these proteins were identified because of their direct interaction with PTB (Figure 1). Remodelling of IRES structures to achieve binding of individual *trans*-acting factors often requires additional proteins^22, 23^ and interestingly, the data show that only when combined with PSF (which was kept constant during the experiment) NONO/p54^nrb^ was able to interact with the IRES RNA (Figure 4a and Supplementary Figure S3A). Although NONO/p54^nrb^ interacts with PTB *in vitro* (Figure 1d), this reaction was found to be partially RNA dependent. Taken together, these data (Figure 4) suggest that NONO/p54^nrb^ interacts with both PTB, and PSF *in vivo*.

To determine whether some of these proteins interacted with endogenous SETD7, cyclin T1, MTG8A and CYP1b1 RNA *in vivo* mRNA immunoprecipitation reactions were carried out (Figure 4b; right-hand panel shows that the proteins were immunoprecipitated as expected, and Supplementary Figure S3B). Cell lysates were incubated with antibodies directed against NONO/p54^nrb^, PSF or PTB and mRNAs that co-immunoprecipitated with these complexes were reverse transcribed and the cDNAs used in qPCR. The data show that all mRNAs were significantly enriched in the complexes that were immunoprecipitated using antibodies directed against, PSF, NONO/p54^nrb^ and PTB compared with controls (Figure 4b and Supplementary Figure S3B).

### Cellular IRES function is regulated by the PTB-containing complex

**Figure 5:**
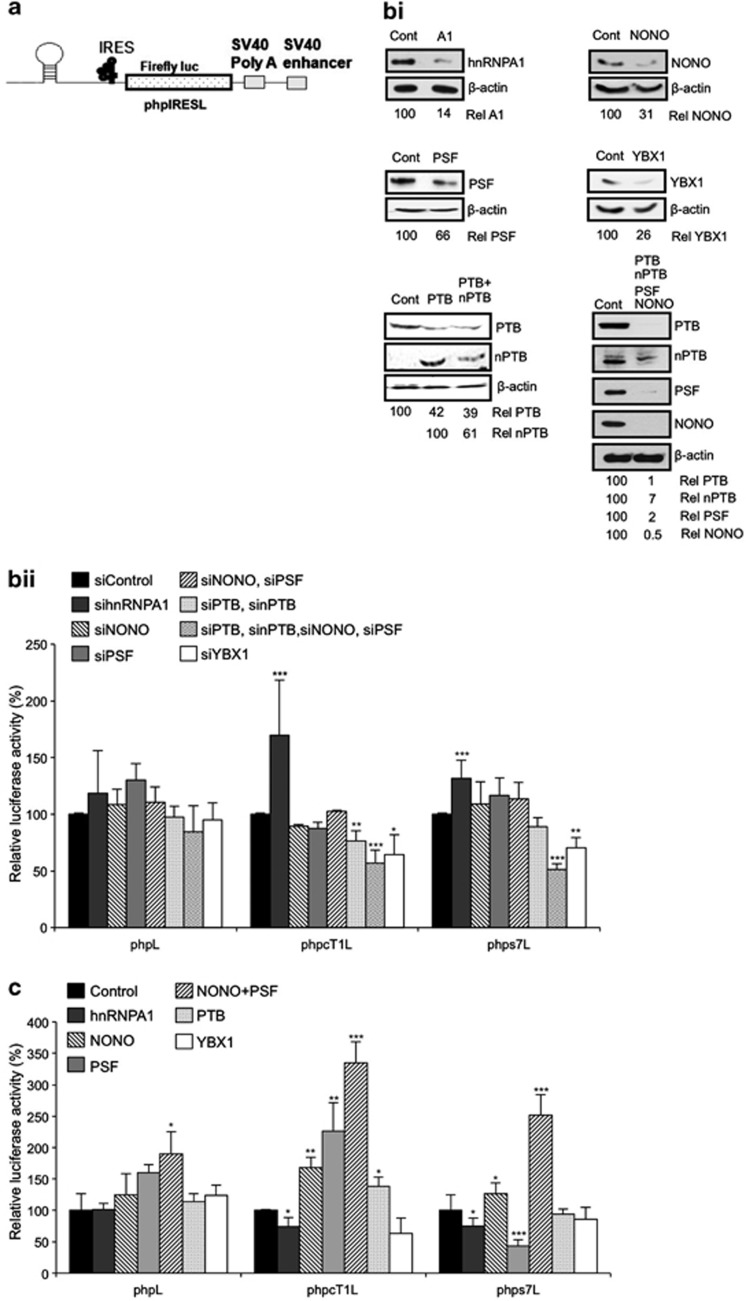
Altering the levels of PTB-interacting proteins effects IRES activity. (**a**) Schematic representation of the monocistronic constructs used in figures **b**ii and **c**, which contains a stable hairpin loop downstream of the 5′ m^7^G cap in order to inhibit cap-dependent translation of the *Firefly* luciferase reporter. (**b**i) HeLa cells were co-transfected with siRNA against the indicated proteins together with the monocistronic reporter plasmid, incubated for 48 h then harvested and western blot analysis was carried out to confirm RNAi success. Immunoblots are quantified relative to *β*-actin levels, and expressed as a percent of levels in the control siRNA treated lysate. (**b**ii) Luciferase assays were carried out on the RNAi lysates. Data are shown relative to a control experiment using control siRNA. Significance (*P<0.05, **P< 0.01 or ***P<0.005) was calculated using an unpaired two-tailed Student's *t*-test (n=3), error bars represent S.D. (**c**) Reticulocyte lysates were primed with 100 ng recombinant protein and 100 ng of *in vitro* transcribed m^7^G capped and polyadenylated reporter RNA, incubated at 30 °C for 90 min, then assayed for luciferase activity. Data are shown relative to a control experiment with no recombinant protein added. Significance (*P<0.05, **P<0.01 or ***P<0.005) was calculated using an unpaired two-tailed Student's *t*-test (*n*=3), error bars represent S.D.

To determine whether these proteins affected IRES-mediated translation from these mRNAs, vectors that contained the cyclin T1 or SETD7 IRESs fused in frame with DNA encoding *Firefly* luciferase and an upstream hairpin to block ribosome scanning (Figure 5a) were transfected into HeLa cells. The levels of hnRNPA1, NONO/p54^nrb^, PSF, PTB and nPTB and YBX1 were reduced and luciferase activity was determined (Figure 5b). The data show that in cells where there is a reduction in PTB and YBX1 expression, IRES function of cyclin T1 was reduced to approximately 70% of the control cell value with a smaller, but significant decrease of SETD7 IRES activity. Decreasing expression of PSF or NONO/p54^nrb^ alone, or in combination, had little or no significant effect on IRES activity. However, when levels of PTB, PSF and NONO/p54^nrb^ were simultaneously reduced, cyclin T1 and SETD7 IRES activity was inhibited about 50% (Figure 5bii). Similar effects were observed with the other apoptotic IRESs tested (Supplementary Figure S4B). A reduction of hnRNPA1 levels was shown to stimulate all the IRESs tested suggesting that this protein functions as an inhibitor of apoptotic IRES activity (Figure 5bii and Supplementary Figure S4B). Little effect was observed when DDX3X, hnRNPA2/B1 or PCBP2 protein levels were reduced (data not shown).

We hypothesize that relocation of IRES-binding proteins to the cytoplasm of apoptotic cells is required to stimulate IRES function. Therefore, to mimic this situation *in vitro*, rabbit reticulocyte lysates were used that were primed with IRES-containing mRNAs and the proteins identified as part of the PTB complex, were added either singly or in combination. Reticulocyte lysates already contain significant amounts of some of these proteins, therefore it was not expected that all of these proteins would give a large stimulation.^24^ However, PSF and NONO/p54^nrb^ and PTB stimulated the cyclin T1 and there was a greater stimulation when PSF and NONO/p54^nrb^ were added in combination (Figure 5c). Interestingly, hnRNPA1 repressed IRES activity with a small, but significant reduction in the amount of luciferase produced (Figure 5c). Similar effects were observed with the SETD7 IRES (Figure 5c) and the other IRESs tested suggesting that the proteins identified are required for IRESs that function during apoptosis (Supplementary Figure S4A).

### Proteins in the PTB-containing complex control the apoptotic rate

**Figure 6:**
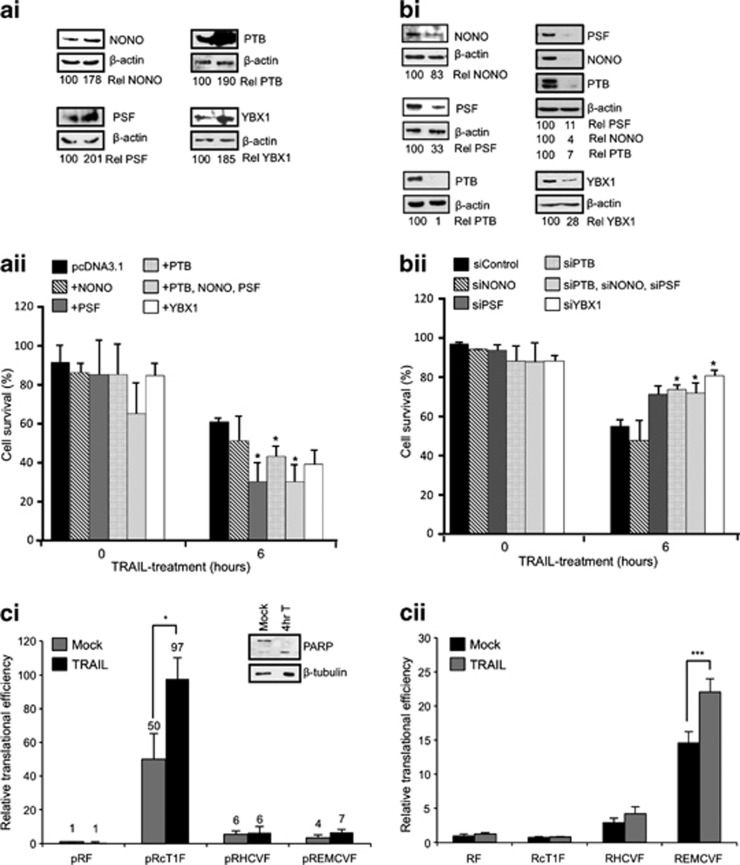
Altered expression of PTB-binding proteins controls apoptotic rates. (**a**) Overexpression of PTB1, NONO/p54^nrb^, PSF either individually or together enhances TRAIL-induced apoptosis. (**a**i) MCF7 cells were transfected with pcDNA3.1, pcDNA3.1-PTB1, pcDNA3.1-NONO/p54^nrb^, pcDNA3.1-PSF, pcDNA3.1-YBX1 plasmids and subjected to 6-h TRAIL treatment. The overexpression was assessed by western blot using antibodies against the proteins indicated. Immunoblots were quantified relative to *β*-actin levels and expressed as a percent of levels in the control cells. (**a**ii) FACS analysis of MCF7 cells overexpressing the proteins above. After TRAIL treatment, cells were stained with Annexin V-FITC and propidium iodide and subjected to FACS analysis. Significance (*P<0.05) was calculated using an unpaired two-tailed Student's *t*-test (*n*=3) relative to the control. (**b**) Depletion of endogenous YBX1, PTB, NONO/p54^nrb^ and PSF individually or in combination protected cells against TRAIL-mediated apoptosis. (**b**i) Western blot of siRNA treated MCF7 cells using antibodies indicated. Immunoblots were quantified relative to *β*-actin levels and expressed as a percent of levels in the control cells. (**b**ii) FACS analysis of siRNA treated MCF7 cells treated as in **a**ii. Significance (*P<0.05) was calculated using a paired two-tailed Student's *t*-test (*n*=3) relative to the control. (**c**i) MCF7 cells were transfected via nucleofection with dicistronic pRIRESF plasmid, incubated for 48 h before mock or TRAIL treatment for 4 h. Both protein and RNA were harvested. *Firefly* luciferase counts were normalized to total protein, and luciferase RNA levels were normalized to GAPDH RNA. The translation efficiency is expressed as *Firefly* luciferase activity/mRNA level. Data are presented relative to this value for pRF in the mock condition. Western blot with *α*PARP was performed to monitor the degree of apoptosis. Significance (*P<0.05) was calculated using an unpaired two-tailed Student's *t*-test (*n*=3), error bars represent S.D. (**c**ii) In all, 1 *μ*g of capped and polyadenylated mRNA was transfected into MCF7 cells, incubated for 2 h before mock or TRAIL-treatment for a further 4 h. Cells were harvested and assayed for both *Renilla* and *Firefly* luciferase. *Firefly* luciferase counts were normalized to *Renilla* luciferase counts and are presented relative to RF in the mock condition. Significance (***P<0.01) was calculated using an unpaired two-tailed Student's *t*-test (*n*=5), error bars represent S.D.

To determine whether the proteins identified within this complex were able to directly influence the rate of apoptosis in cells treated with TRAIL, cells were transfected with vectors allowing for the overexpression of NONO/p54^nrb^, PSF and PTB or with the corresponding siRNAs to reduce their expression (Figures 6ai and bi). These cells were then incubated with TRAIL for 6 h, harvested and stained with Annexin V and propidium iodide before being subjected to flow cytometry to determine the degree of apoptosis. Following exposure to TRAIL, 40% of the control cells underwent apoptosis at 6 h. PSF, PTB or PTB+PSF+NONO/p54^nrb^ overexpression resulted in a greater degree of apoptosis, 6 h after treatment with TRAIL (Figure 6aii). In contrast, depletion of PSF, PTB or PTB+PSF+NONO/p54^nrb^ reduced cell death with 15–20% apoptosis occurring compared with 50% observed in the control cells (Figure 6bii). These data illustrate that altering the levels of these RNA-binding proteins alone is sufficient to modulate apoptosis rates and strongly suggest that the PTB-containing complex is important for this process to proceed efficiently.

### The PTB-containing complex interacts with the cyclin T1 IRES in the nucleus

We have shown previously that certain cellular IRESs, in contrast to viral IRESs such as encephalomyocarditis virus (EMCV) and hepatitis C virus (HCV), are inactive when IRES-containing RNAs are transfected into the cytoplasm and require a ‘nuclear event' before they are fully functional.^15^ Our data show that a ‘remodelled' PTB-containing complex is present in both the nucleus and the cytoplasm of apoptotic cells. To test whether there was nuclear activation of IRESs in apoptotic cells rather than direct cytoplasmic activation, DNA constructs and *in vitro* transcribed reporter RNAs containing the cyclin T1 IRES were used. As controls, RNAs that contained the HCV IRES, which binds PTB, but is not dependent on PTB for function^10^ and the EMCV IRES that binds to PTB and whose activity is stimulated by this protein^25, 26^ were used. There was activation during apoptosis of the EMCV IRES with both RNA and DNA transfection (presumably because the levels of PTB that activates this IRES increase in the cytoplasm in apoptotic cells; Figure 1a). There was no stimulation of HCV IRES activity, as expected, and the cyclin T1 IRES was only activated following DNA transfection (Figure 6c).

**Figure 7:**
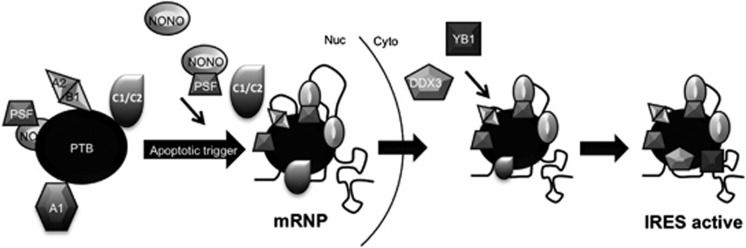
A model to illustrate how formation of a nuclear mRNP containing the PTB complex could be required for cellular IRES activity. Our data suggest remodelling of a PTB-containing complex occurs following treatment with the apoptosis-inducing ligand TRAIL. Thus, the IRES-inhibitory protein hnRNPA1 decreases in association with PTB, whereas the IRES-stimulatory proteins NONO/p54^nrb^, PSF and hnRNPA2/B1 increase in association. This complex then binds to the IRES RNA, and shuttles to the cytoplasm as an mRNP. Here, further IRES-stimulatory proteins including DDX3X and YBX1 are recruited to the complex, which is then competent to stimulate apoptotic IRES-mediated translation

Therefore, we hypothesize that the complex that contains PTB is remodelled in the nucleus of apoptotic cells and interacts with IRESs in cellular mRNAs that encode proteins that function during apoptosis, providing the ‘nuclear event'. In the cytoplasm, recruitment of additional factors are necessary to permit a fully functional mRNA protein complex that is recruited to the polysomes and translation is initiated (Figure 7).

## Discussion

It is clear from the study of a number of systems, that mRNA-binding proteins have a crucial role in the post-transcription regulation of gene expression. For example, HuR and its neuronal relatives (HuB, HuC and HuD) associate with numerous mRNAs encoding proteins with a wide range of cellular roles including division, survival, immune response and differentiation, and influence their stability and synthesis.^27^ HuR does not act in isolation and interplay of HuR with other proteins and miRNAs is necessary to obtain specificity of interaction with these mRNAs.^28^

Similarly, PTB is also known to interact with mRNAs that encode proteins with a diverse range of functions^9^ and some of its binding partners have been identified.^13^ However, the full complement of mRNA-binding proteins with which it associates has not been previously addressed. We therefore examined the PTB-containing complexes that form in control and apoptotic cells and addressed whether they interact with RNA elements present in mRNAs that are translated during apoptosis (Figures 1 and 2, Supplementary Figures S2–S4). Our data strongly suggest that a PTB-containing complex is remodelled during TRAIL-mediated apoptosis to form one that contains PSF, YBX1, NONO/p54^nrb^, hnRNPA2/B1, hnRNPC1/C2 and DDX3X (Figures 1a–c). Interestingly, some of these proteins have been shown previously to be involved in the translation of mRNAs that contain IRES elements.^29, 30, 31^ The data also suggest that remodelling of this complex during apoptosis is achieved, in part, by a relocalization of these proteins, with PTB, YBX1, NONO/p54^nrb^, hnRNPA2/B1 and hnRNPC1/C2 showing an increase in their cytoplasmic localization during TRAIL-mediated apoptosis (Figure 2ai), whereas hnRNPA1 that has been shown to inhibit the translation of the Apaf-1 IRES^32^ becomes more nuclear in its localization (Figure 2ai).

Using RNA affinity-based approaches, we show that proteins in the PTB-containing complex interacted with the highly active cyclin T1 and SETD7 IRESs (Figures 3 and 4), in addition to those in MTG8a and cyp1b1 (Supplementary Figures S2–S3). Moreover, either singly or in combination they affect IRES activity both positively and negatively (Figure 5, Supplementary Figure S4). Importantly, we show that by modifying the expression of these proteins alone, it is possible to control cell fate (Figures 6a and b). Thus, overexpression of the proteins that form part of the PTB-containing complex increases the rate of apoptosis while reducing their expression delays this process (Figures 6a and b). Given the number of protein factors that are required, these cellular IRESs clearly differ markedly from viral IRESs that require relatively few, or in some cases, no *trans*-acting factors.^33^ This is probably due to inherent differences in the stability of the structures formed by viral IRES RNAs compared with cellular IRES mRNAs, and in turn this is likely to reflect the very different circumstances under which these types of motifs are used to initiate translation. The data suggest that protein complexes act as chaperones to remodel the IRES RNAs into structures that are able to recruit the ribosome, whereas, in contrast, highly structured viral IRESs are already competent in this regard.^34^ For example, compactly folded IRESs found in the IGR of the dicistroviridae family are able to bind the 40S subunit via stable pseudoknots, with the first pseudoknot occupying the P-site such that the 40S subunit is positioned correctly for elongation.^35^ Moreover, as initiation of translation on mRNAs that contain cellular IRESs can also frequently occur via scanning (e.g., c-Myc is translated by both mechanisms),^15^ and cellular IRESs are not functional during all physiological situations, these elements, by necessity, are more likely to have flexible structures that require chaperones for activity.^34^ Interestingly, in yeast and *Drosophila* there is an inverse correlation between IRES function and structure.^36^ The lack of structural conservation, even between IRESs that are found in mRNAs that have similar functions (e.g., the Myc family of IRESs), suggests that they have evolved in a very different way from viral IRESs.^34, 37, 38, 39^ Therefore, regulating the recruitment of specific subsets of RNA-binding proteins to remodel the structure of cellular IRES into an ‘active mode' would permit the control of translation of the corresponding mRNAs as, and when, required. Controlling the bioavailability of these factors or the formation of complexes in which they reside, would directly regulate cytoplasmic control of gene expression in the absence of transcriptional control. Taking these factors into account, it is perhaps unsurprising that IRES-mediated translation on cellular mRNAs is a very complex process requiring many RNA-binding proteins, in addition to some canonical initiation factors.^20, 30, 34, 40^

We hypothesize, therefore, that for full activity it is necessary for the IRES elements to associate with the PTB-containing complexes in the nucleus (Figure 7) in support of previous data, which show that many cellular IRESs require a ‘nuclear event' before they are fully active.^15^ It is interesting to note that many of the proteins identified also function in splicing^41^ and one possibility is that these proteins become associated with the IRES-containing mRNA during splicing. Indeed, many factors that have a primary role in splicing have also been shown to directly regulate translation. For example, SFRS1 promotes mRNA association with polysomes by modifying 4EBP1 activity.^42, 43^

In summary, our data suggest a model whereby altering the repertoire of mRNA-binding proteins present in a PTB-containing complex in the nucleus of apoptotic cells allows their interaction with specific mRNAs, and influences their translation thus permitting post-transcriptional control of gene expression.

## Materials and Methods

### Plasmid constructs

The dicistronic constructs RcT1F, Rset7F and R1b1F are described in Bushell *et al.*^2^ RMTG8F is described in Mitchell *et al.*^22^ The monocistronic constructs were cloned from the dicistronic constructs using *Spe*I and *Nco*I restriction enzymes and cloned to phpL.^40^ The mammalian expression constructs of NONO/p54^nrb^, PSF and PTB in pcDNA3.1-vector have been described in Cobbold *et al.*^30^

### Protein expression

Recombinant hnRNPA1, NONO/p54^nrb^, PCBP1, PCBP2, PTB and YBX1 were expressed and purified as described.^30^

### Cell culture, TRAIL treatment and transfections

MCF7 and HeLa cells were maintained in DMEM supplemented with 10% FBS and 2 mM L-glutamine. Cells were treated with 0.5 *μ*g/mlTRAIL (PeproTech, Rocky Hill, NJ, USA) for times indicated in the figure legends and harvested to produce either whole cell, or nuclear and cytoplasmic lysates.

### Plasmid DNA and mRNA transfections

DNA transfections in HeLa cells were carried out using Lipofectamine 2000 (Invitrogen, Carlsbad, CA, USA) following the supplier's instructions. DNA transfections in MCF7 cells were performed using nucleofector AmaxaV kit (Lonza, Köln, Germany), using 2 × 10^6^ cells/1.5 *μ*g plasmid/transfection following the supplier's instructions.

### Reporter assays

The activity of *Firefly* luciferase (monocistronic reporter vectors) or *Firefly* and *Renilla* luciferases (dicistronic reporter vectors) in lysates prepared from transfected cells were measured using a Luciferase Reporter Assay System or with Dual-Luciferase Reporter Assay System (Promega, Madison, WI, USA), and light emission was measured over 10 s using an OPTOCOMP I luminometer (MGM Instruments, Hamden, CT, USA).

### RNA interference

Dharmacon (Waltham, MA, USA) on-target pre-designed siRNA were used to knockdown levels of NONO/p54^nrb^, PSF, YBX1 and hnRNPA1. Levels of PTB1 and nPTB were depleted using siRNAs of sequences: CUUCCAUCAUUCCAGAGAA.dT.dT and GAGAGGAUCUGACGAACUA.dT.dT. Dharmacon control siRNA (C3; UGGUUUACAUGUUUUCUGA) was used in control transfections. Transfections were carried out in 24-well plates or 15 cm plates using either Dharmafect duo (for dual siRNA and reporter plasmid transfections of HeLa cells) or Amaxa nucleofection (for siRNA-mediated knockdown in MCF7 cells). Cells were harvested after 48 or 72 h in the case of YBX1 knockdown cells.

### Preparation of cytoplasmic and nuclear lysates

Cells were pelleted by centrifugation at 4 °C at 250 × *g* for 2 min, then resuspended in (10 mM HEPES pH 7.9, 10 mM KCl, 0.1 mM EDTA, 0.1 mM EGTA, 1 mM DTT, 50 mM sodium fluoride, 50 mM glycerol-2-phosphate, 1 × protease inhibitor and 0.5% NP40). Samples were centrifuged to obtain cytoplasmic fraction. The nuclei pellet was resuspended in (20 mM HEPES pH 7.9 400 mM NaCl, 1 mM EDTA, 1 mM EGTA, 1 mM DTT, 50 mM sodium fluoride, 50 mM glycerol-2-phosphate, 1 × protease inhibitor) and lysates generated.

### Western blot and antibodies

Membranes were probed with the following antibodies: *β*-actin (Sigma-A5441; Sigma Aldrich, Gillingham, UK); cyclin T1 (Abcam-755105, Cambridge, UK); DDX3X (Abcam-37160); GAPDH (Cell Signalling-2118; Cell Signalling Technology, Danvers, MA, USA); hnRNPA1 (Abcam-4791); hnRNPA2/B1 (Abcam-6102); hnRNPC1/C2 (Abcam-10294); Lamin A/C (Cell Signalling-2032); NONO/p54^nrb^ (Abcam-70335); PARP (Cell Signalling-9532); PCBP2 (Abcam-96169); PSF (Abcam-99357); PTB (BB7), gift from Professor C Smith, University of Cambridge; nPTB (in house); RPS6 (Cell Signalling-2217); SETD7 (Cell Signalling-2825); and YBX1 (Abcam-12148).

### Immunoprecipitation with *α*-PTB

Protein A/G beads were pre-bound to 500 *μ*l monoclonal anti-PTB antibody (BB7) and incubated with cell extracts. Proteins were eluted in SDS-PAGE loading dye. For the RNase-treated samples, an additional incubation in 10 *μ*g/ml RNase A at 37 °C for 30 min was included before protein elution.

### Confocal microscopy

MCF7 were seeded onto 13 mm coverslips then treated with 0.5 *μ*g/ml TRAIL for 3 h. Cells were fixed using 4% paraformaldehyde and permeabilized using 0.1% Triton-X-100. Incubation with primary BB7 anti-PTB antibody was carried out at a concentration of 1 : 1000, followed by incubation with fluorophore-conjugated anti-mouse secondary antibody at a concentration of 1 : 200. Finally, cells were incubated in Hoechst stain for 1 h at RT, mounted and viewed on a Zeiss confocal microscope (Zeiss, Cambridge, UK).

### FACS analysis

In all, 1 × 10^6^ MCF7 cells were transfected with either non-targeting siRNA or siRNA targeted to PTB1/nPTB, PSF, NONO/p54^nrb^, YBX1 or hnRNPA1. After 48 h, cells were treated with 0.5 *μ*g/ml TRAIL over a 0–6 h time course. Cells were trypsinized and stained with Annexin FITC and propidium iodide. Cells were then subjected to FACS analysis on a BD Biosciences FACSCalibur (BD Biosciences, Oxford, UK).

### RNA affinity purification

Briefly, biotinylated RNA was incubated with HeLa cell lysates (4C, Mons, Belgium) and the complexes purified on streptavidin-conjugated magnetic beads (Invitrogen). Eluted proteins were precipitated using 10% TCA and solubilized in SDS-PAGE loading dye (see Figure legend 3a for further details).

### Immunoprecipitation using purified proteins

In all, 100 *μ*l A/G sepharose beads bound to monoclonal anti-PTB antibody (BB7) were used to immunopreciptate PTB protein and its interacting partners that were incubated in an equal molar ratio. The beads were pelleted by centrifugation and after extensive washing proteins eluted in SDS-PAGE loading dye.

### Filter-binding assays

Approximately 23 000 c.p.m. of labelled transcript was added to 10 *μ*l of buffer mix containing 2 *μ*l of 5 × transcription buffer (200 mM Tris-HCl [pH 8.0], 40 mM MgCl_2_, 10 mM spermidine, 250 mM NaCl) 0.75 *μ*l of 1 M DTT, 2 *μ*l of tRNA (10 mg/ml), 1 *μ*l of 10 mM rATP, and 40 U of RNasin. Recombinant proteins between the range of 0.050 and 2 *μ*g were then incubated with the mixture at room temperature for 10 min. Reaction mixtures were filtered through a nitrocellulose membrane, the membranes were washed, air dried and counted in a scintillation counter. Values for dissociation constants were determined from the scintillation readings, whereby the angle of the slope of the graph is equal to 1/*K*_d_.

### UV-crosslinking

In all, 50 000 c.p.m. of ^32^P-CTP-labelled RNA was used with 200 nM of protein in a final volume of 20 *μ*l (10 mM HEPES pH 7.4, 3 mM MgCl_2,_ 100 mM KCl, 5 mM creatine phosphate, 1 mM DTT, 1 mM ATP, 6% glycerol and 0.1 *μ*g/*μ*l tRNA) were incubated at room temperature for 15 min with or without 1 × , 5 × or 10 × molar excess of cold competitor RNA (nonspecific competitor was transcribed from *Firefly* luciferase gene). In total, 0.05 *μ*g/*μ*l heparin was added and incubated for a further 15 min. The samples were subjected to UV radiation of 275 J/m^2^ for 30 min on ice. RNase cocktail (0.1 mg/ml RNase A, 0.1 mg/ml RNase T1, 0.1 mg/ml RNase V1) was added and incubated at 37 °C for 30 min before SDS-PAGE loading dye was added, samples were run on a SDS-PAGE gels, fixed, dried and exposed to a phosphorimager screen (PhosphorImager, Storm, GE Healthcare, Chalfont St Giles, UK).

### RNA immunoprecipitations

These were carried out as described previously.^30^ More details can be found in the figure legend of Figure 4b.

### Reverse transcription and qPCR

Reverse transcription was carried out using 5 *μ*g RNA and with either oligo(dT) or random priming using Superscript III Reverse Transcriptase System using standard protocol (Invitrogen). QPCR was carried out in a 50 *μ*l reaction containing 5 pmol/*μ*l of each primer, 25 *μ*l Syber Green master mix (Qiagen, Hilden, Germany) and 0.5 *μ*l cDNA in each optical tube. Reactions were carried out in an Applied Biosystems Real-Time PCR machine (Applied Biosystems, Cheshire, UK). Primers to cDNAs were designed according to sequences from the NCBI database and relative quantification was made against IgG control bound beads. All PCR reactions were carried out in triplicate. Primers (5′–3′) used for cyclin T1, (F) CCGAGAGCACAAAGAAAAGC and (R) GGCTACTATGTTTTGGATCACCAGG; SETD7, (F) GAAGATTGCCCACCAAAGGAACTG and (R) GCAAGCCATCTTTCTCCTGAAGAC; MTG8, (F) GCCCAGCAGCAGGGAGACAC and (R) GAAGGGGTTCCCGGGGTGGT; CYP1b1, (F) CAGCCCACTCAGCCCACTGC and (R) AGAGGTGGTGGCCGGGGTTT; actin, (F) CGACAACGGCTCCGGCATGT and (R) TGGGCCTCGTCGCCCACATA; *Firefly* luciferase, (F) ATGGAAGACGCCAAAAACAT and (R) GCCTTATGCAGTTGCTCTCC.

### *In vitro* transcription and translation reactions

Capped mRNA was generated using with the RiboMAX Large Scale Production System-T7 (Promega) and poly-A tailed with Poly(A) tailing Kit (Ambion, Austin, TX, USA) according to the manufacturer's instructions. *In vitro* translation reactions were performed using the Flexi rabbit reticulocyte lysate system (Promega). Reactions contained 100 ng capped RNA template in a final volume of 12.5*μ*l. Protein factors were added at 80–150 nM. Reactions were incubated at 37 °C for 1.5 h before measurement of luciferase activity.

### Mass spectrometry

Samples were resolved via SDS-PAGE and sliced into 1–1.5 mm slices, before being subjected to in-gel trypsinolysis and analyzed via nanoLC-MS/MS as described previously.^44^ Protein and peptide identification were carried out using the MASCOT programme and validated using scaffold (Proteome Software Inc., Portland, OR, USA).

## Abbreviations

EMCV: encephalomyocarditis virus
HCV: hepatitis C virus
IRES: internal ribosome entry segment
PTB: polypyrimidine tract-binding protein
TRAIL: TNF-related apoptosis inducing ligand
