# Supplementary material for: Remodelling of a polypyrimidine tract-binding protein complex during apoptosis activates cellular IRESs
Source: Cell Death Differ. 2013 Oct 18;21(1):161–71. doi: 10.1038/cdd.2013.135 (PMC3857619; doi:10.1038/cdd.2013.135)
Supplement: Supplementary Information [file cdd2013135x1.pdf]

(S1Ai)

Protein	Cytoplasmic Mock	Cytoplasmic TRAIL	Nuclear Mock	Nuclear TRAIL
PTBP1	5	9	15	13
ATX1		1		1
DDX1	1	1		
DDX21				1
DDX3X	2	3		1
DDX5	1		5	3
eIF2a	1			
eIF4A3			1	1
eIF4G1	1			
F120A		2		
HNRGT				1
hnRNPA1			5	2
hnRNPA2/B1			2	4
hnRNPA3				1
hnRNPC1/C2			1	3
hnRNPD0	1		1	
hnRNPF				1
hnRNPG				1
hnRNPK			3	
hnRNPL				1
hnRNPM	1		1	
hnRNPR				1
hnRNPU				1
MATR3			12	6
MINT	1	1		
NONO/p54^{nrb}	1	1	2	2
NPM			2	3
NUCL			2	1
PA2G4		1		
PABP1	1			
PAIRB	2	3		
PCBP2	1	1		
PSF/SFPQ			4	2
RAVER1		1		
RUXG				1
YBX1	2	2		1
ZRAB2	1		1	1

(S1Aii)

Protein	Cytoplasmic Mock	Cytoplasmic TRAIL	Nuclear Mock	Nuclear TRAIL
R13AX	1			
RPL13	3	3		1
RPL14	1	1		
RPL18	1	1		
RPL22	2		1	
RPL3	1			
RPL4		3		
RPL5	2			
RPL6	1			
RPL7	1	1		
RPL8	1			
RPLA0	3	3		
RPLA2	1			
RPS10	1			
RPS13		1		
RPS18	4	1	1	
RPS19	1			
RPS2	2			
RPS20	1			
RPS25	1			
RPS3	5	4	1	
RPS3A	3			
RPS4X	2	1		
RPS6	2	1		
RT33	1	1	1	

Protein identification probability

(S1Aiii)				
	>95%	80-94%	50-79%	20-49%

(S1B)

	Cytoplasmic		Nuclear	
	**Mock**	**TRAIL**	**Mock**	**TRAIL**
NONO			1	1.1 (±0.1)
PSF			1	0.9 (±0.03)
hnRNPA1			1	1.5 (±0.18)
hnRNPC1/C2			1	1.4 (±0.06)
DDX3X	1	2.5 (±1.27)		
YBX1	1	1.5		
hnRNPA2/B1			1	1 (±0.22)

Supplementary Figure S1: Additional proteins identified in the proteomic analysis of the PTB immuno-precipitation. MCF7 cells were mock or TRAIL-treated for 3 hours, then harvested to produce nuclear and cytoplasmic lysates. These lysates were then used in a PTB-immuno-precipitation, the proteins eluted from the beads and identified by LC-MS/MS. Proteins were categorised into functional groups including **(Ai)** RNA-binding proteins and **(Aii)** ribosomal and ribosomal-associated proteins, according to the NCBI database, number of unique peptides are displayed. **(Aiii)** Key to protein identification probability for the proteins identified.
(B) Densitometric quantification of western blots in Figure 1Ci. Each value was normalised against corresponding PTB value (Mock/Trail) and the Mock of each PTB partner is set to 1. (STDEV of 3 independent IP experiments.)

(S2Ai)

Protein	GAPDH	MTG8a	CYP1b1	CyclinT1	SetD7	Apaf-1
CSTF2				2	1	1
DAZP1				4		
DDX1				5	1	1
DDX17						5
DDX21				2	2	7
DDX39			1	1		
DDX3X		1	1	2	1	4
DDX5				4	2	17
DHX29				1	2	1
DHX30				4		
DHX40			1	2	2	1
DHX57				2	1	1
DHX9				12	2	8
DRBP1				2	1	
DSRAD				2		
E2AK2				2		1
ECP				1	1	
EF1A1	7	5	6	5	6	2
EF1A2			1			
EF1D	1	2	1	7	4	
EF1G	1	3	3	1		
EF2	1	4	1	8	1	
eIF3j			1			
eIF3g				1		
eIF3c				1		
eIF3f			1	1		
eIF4B		3	2		1	1
eIF5A		1		1		
eIF5B		1			1	1
ELAV1					2	2
EWS	1			2	1	5
FMR1				3	4	
FUBP1	2	1		14	2	
FUBP2				10		
FUBP3				2		
FUS/TLS	3			2	2	4
FUSIP					1	1
FXR1				3		
FXR2	1			3	3	3
GEMI5					3	
hnRNPA0						1
hnRNPA1				4	3	7
hnRNPA2/B1		1	2	6	5	7
hnRNPA3	1			1		3
hnRNPC1/C2	1	3	1	1		6
hnRNPD				2		
hnRNPG				3	3	1
hnRNPH1				2	1	4

Protein	GAPDH	MTG8a	CYP1b1	CyclinT1	SetD7	Apaf-1
hnRNPH3				1	1	
hnRNPK	1	2	2	8	1	3
hnRNPM	2	1	1	17	11	17
hnRNPQ				4	1	
hnRNPRL1				2		5
hnRNPU				6	2	3
IF2B2					1	
IMP3					1	1
LARP1			1	3		
LARP4						2
M10L1	1	1			2	1
MORC3				1	2	1
NOLA2						2
NONO/p54^{nrb}	1	1	2	7	12	17
NUFP2				7		2
PABP1		1	1	6	3	3
PAIRB	2	3	7	6	4	4
PCBP1	1	1	3	10	2	
PCBP2	1	1	2	3		
PRP31			1	1		
PRP8			2	2		
PTBP1		4	1	2	1	4
RBM8A				1		1
RBM10				1	2	2
RBM14						1
RBM27				1	1	
RNPS1	1	2			1	
RU2A	1					
RU2B	1					2
SAM68					1	3
SF3A2			1			
SF3B1		1		1	2	
SF3B2						3
SF3B3			1	2	1	
SF3B4				1		
SFPQ (PSF)	3		6	7	3	20
SFRS1						2
SFRS2		3		1	1	1
SFRS7	2	1	1	1	1	2
SMD1				1		
SMD2	3	2				3
SNRPA	2			1		2
SPF45				1		
TIAR				5		
U3IP2					2	
YBOX1	5	7	8		3	3
ZCC2				5	1	1

(S2Aii) **Protein identification probability**

>95%	80-94%	50-79%	20-49%

Supp S2A

(S2B)

Protein	GAPDH	MTG8a	CYP1b1	CyclinT1	SETD7	Apaf-1
BOP1			1			1
RPL10		3		2	2	2
RPL10A		1	1	2	3	3
RPL11	1	1	1	1	1	1
RPL12	1	4		7	5	6
RPL13	1	3	1	2	2	6
RPL13A				1	3	
RPL14	1	2	1	1	1	3
RPL15				1	3	1
RPL17	1	2		3		5
RPL18	1	4	2	5		2
RPL18A				2		2
RPL19	1	1	1	2	2	2
RPL21				2		5
RPL22	2	2		4	1	2
RPL23				2	1	3
RPL23A	3	1		4	2	4
RPL24	1	2	1	3		4
RPL26		1		4	3	
RPL27				1	1	4
RPL27A					1	1
RPL28		1		2		2
RPL29	1	1		1	1	2
RPL3				6		
RPL30	1	1	1	2	1	2
RPL31	1	2		1		3
RPL32	1					3
RPL34		1		2	1	2
RPL35		1		1		1
RPL35A				1	3	1
RPL36		1		1	2	1
RPL36A		1				1
RPL37A						2
RPL38				2	1	1
RPL4	7	8	8	3	4	4
RPL5		1		5	5	
RPL6	1	1	3	3	5	3

Protein	GAPDH	MTG8a	CYP1b1	CyclinT1	SETD7	Apaf-1
RPL7	1	1		4	4	3
RPL7A	2	4	3	4	2	3
RPL8		1	1	4	3	2
RPL9				2	1	1
RPLA0	4	4	3	10	7	12
RPLA1				1	1	
RPLA2				2	3	
RPS10	2	2		4	2	2
RPS12				1	2	3
RPS13		3		2	4	4
RPS14	1			4		6
RPS15						1
RPS15A	1			1	4	3
RPS16				2	2	3
RPS17		1		4	2	6
RPS18		4		6	3	4
RPS19	2	2		7	2	8
RPS2	1	1	1	1	4	4
RPS20	1	2		2		5
RPS21				1		
RPS24		1			2	3
RPS25	1	2		1	1	3
RPS26		1			1	2
RPS27				2	1	2
RPS28					1	
RPS3		1	1	3	8	6
RPS30						1
RPS3A	2	2	4	3	4	5
RPS4X		1		3	6	4
RPS5		3		3	3	3
RPS6	3	1	2	5	3	2
RPS7				4		2
RPS8	1	2	2	1	2	2
RPS9				2	1	3
RPSSA	4	4	4	7	6	5
RRBP1	5	8	18	19	9	2

(S2Ci)

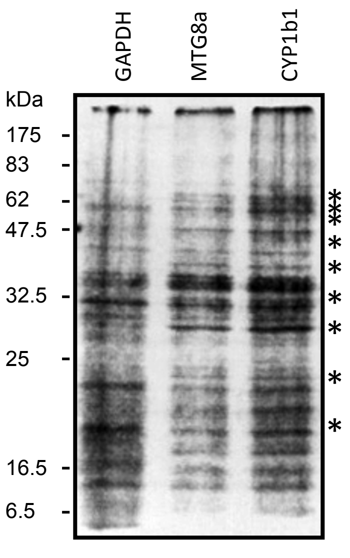

(S2Cii)

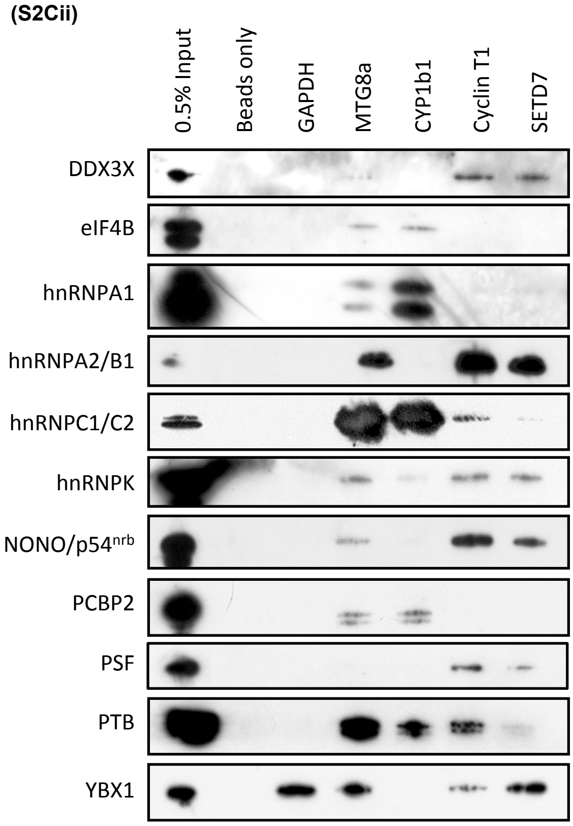

<u>Supplementary Figure S2: An additional two apoptotic IRES elements were screened for binding proteins.</u>

(Ai) Complete list of all the RNA-binding proteins (as specified in the NCBI database) identified as binding to the five apoptotic IRESs and the GAPDH control. Numbers displayed are the number of unique peptides identified per protein. **(Aii)** Key to protein identification probability for the proteins identified.

(B) As Ai, the complete list of all the ribosomal proteins and ribosomal binding proteins (as specified in the NCBI database) identified as binding to the five apoptotic IRESs and the GAPDH control.

(Ci) Proteins were eluted from the bait RNA by boiling for 5 minutes in 0.1% SDS then precipitated, washed, and resuspended in SDS-PAGE loading dye, before being resolved on SDS-PAGE. The gels were stained using Colloidal coomassie. Bands unique to one or more of the IRESs screened compared to control (GAPDH) are indicated with arrows. **(Cii)** To complement the mass spectrometry data, the SDS-elutions from the RNA-affinity experiments were analysed via immunoblotting for identified RNA-BPs.

(S3A)

**Protein**	**MTG8a**	**CYP1b1**
DDX3X	No binding	No binding
hnRNPA1	4.6 [±0.7] x 10^{-6}M	No binding
hnRNPA2	4.9 [±1.0] x 10^{-7}M	6.4 [±2.2] x 10^{-6}M
hnRNPK	3.1 [±1.2] x 10^{-6}M	5.4 [±0.1] x 10^{-7}M
NONO	No binding	No binding
PCBP2	8.1 [±2.1] x 10^{-6}M	1.3 [±0.4] x 10^{-6}M
PSF	3.0 [±1.8] x 10^{-6}M	6.5 [±2.8] x 10^{-7}M
NONO (+PSF)	2.1 [±0.6] x 10^{-6}M	4.4 [±0.9] x 10^{-7}M
PTB	1.7 [±0.3] x 10^{-6}M	3.2 [±1.3] x 10^{-6}M
YBX1	3.7 [±2.1] x 10^{-7}M	5.6 [±1.1] x 10^{-7}M

(S3B)

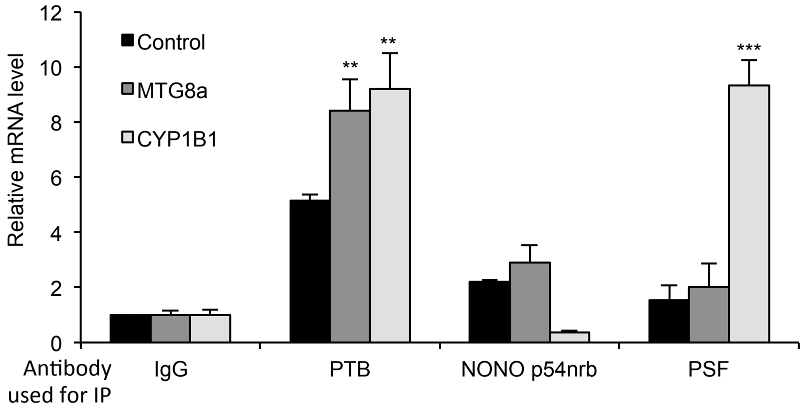

Supplementary Figure S3: *In vitro* binding studies with two additional apoptotic IRES-RNAs.

(A) Dissociation constants were calculated for each protein/RNA combination for the two additional apoptotic IRESes using filter binding assays. Standard deviation is shown in square brackets, and the data is the average of three repeats.

(B) Endogenous RNA-BP binding to IRES-RNA was shown using RNA-IP with antibodies against a specific RNA-BP, followed by qRT-PCR using primers specific for MTG8a, CYP1b1 or control RNA. Results are shown relative to a non-specific IgG immunoprecipitation. The data show enrichment of IRES RNA bound by these proteins. Significance ($P<0.05$, $P<0.01$ or $P<0.005$) was calculated using a unpaired two-tailed Student’s *t*-test for the quantity of IRES RNA versus the control RNA in each IP. Error bars represent standard deviation, and the data presented is the average of three repeats.

(S4A)

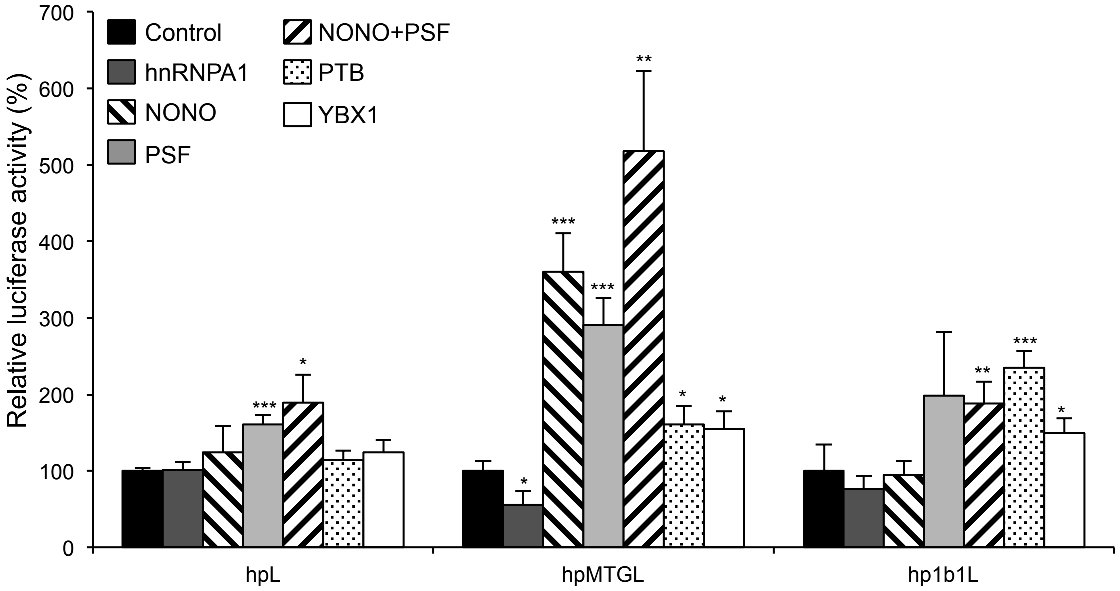

(S4B)

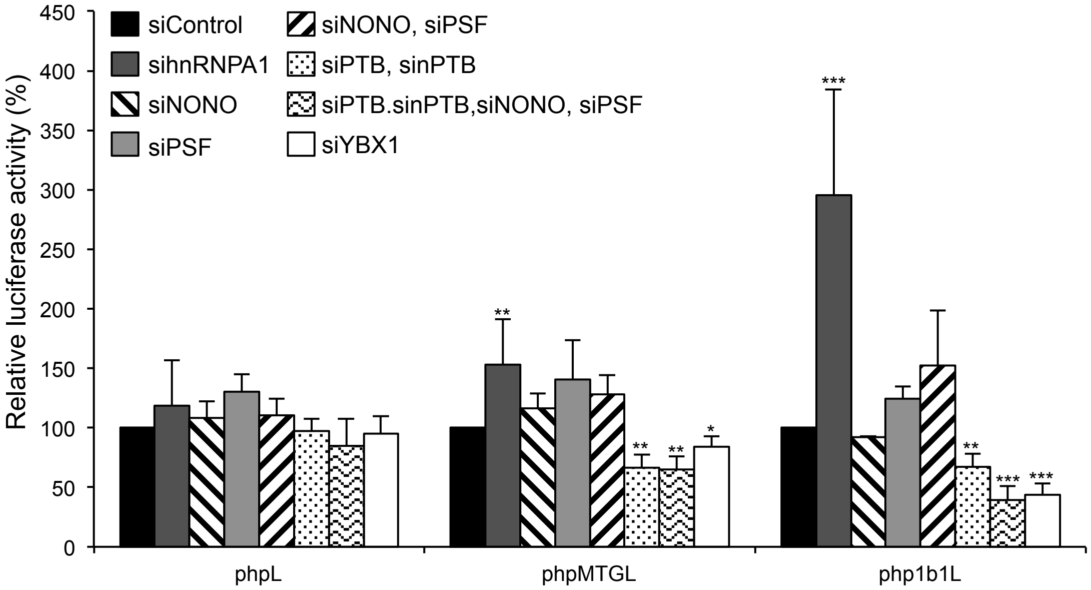

<u>Supplementary Figure S4: Functional assays with two additional apoptotic IRES-RNAs.</u>

(A) Retic lysate was primed with 100ng recombinant protein and 100ng *in vitro* transcribed m^7G capped and polyadenylated reporter RNA, incubated at 30ºC for 90 minutes, then assayed for luciferase activity. Addition of hnRNPA1 inhibited the activity of both IRESes, whereas addition of NONO, PSF, NONO+PSF, PTB or YBX1 stimulated one or both IRESes. Data are shown relative to a control experiment with no recombinant protein added. Significance (P<0.05, P<0.01 or P<0.005) was calculated using an unpaired two-tailed Student's *t*-test for luciferase activity from each reporter in the control, no protein addition, versus protein addition. Error bars represent standard deviation, and the data presented is the average of three repeats.

(B) HeLa cells were co-transfected with siRNA against the indicated proteins together with the monocistronic reporter plasmid. Cells were incubated for 48 hours before harvesting, then assayed for luciferase expression. The data show that depletion of hnRNPA1 stimulates the activity of both IRESes, whereas depletion of PTB+nPTB, PTB+nPTB+NONO+PSF or YBX1 inhibited IRES activity. Data are shown relative to a control experiment using control siRNA. Significance (*P<0.05, **P<0.01 or ***P<0.005) was calculated using an unpaired two-tailed Student's *t*-test (n=3), error bars represent standard deviation.
